# Habitual Diets Are More Expensive than Recommended Healthy Diets

**DOI:** 10.3390/nu15183908

**Published:** 2023-09-08

**Authors:** Manoja P. Herath, Sandra Murray, Meron Lewis, Timothy P. Holloway, Roger Hughes, Sisitha Jayasinghe, Robert Soward, Kira A. E. Patterson, Nuala M. Byrne, Amanda J. Lee, Andrew P. Hills, Kiran D. K. Ahuja

**Affiliations:** 1 School of Health Sciences, College of Health and Medicine, University of Tasmania, Launceston, TAS 7248, Australia; manoja.herath@utas.edu.au (M.P.H.); sandra.murray@utas.edu.au (S.M.); timothy.holloway@utas.edu.au (T.P.H.); sisitha.jayasinghe@utas.edu.au (S.J.); robert.soward@utas.edu.au (R.S.); nuala.byrne@utas.edu.au (N.M.B.); andrew.hills@utas.edu.au (A.P.H.); 2 School of Public Health, Faculty of Medicine, The University of Queensland, Herston, QLD 4006, Australia; m.lewis@uq.edu.au (M.L.); amanda.lee@uq.edu.au (A.J.L.); 3 School of Health Sciences, Swinburne University of Technology, Melbourne, VIC 3122, Australia; rmhughes@swin.edu.au; 4 School of Education, College of Arts, Law and Education, University of Tasmania, Launceston, TAS 7250, Australia; kira.patterson@utas.edu.au; 5 Nutrition Society of Australia, Crows Nest, NSW 1585, Australia

**Keywords:** food cost, food prices, affordability of food, Northwest Tasmania, regional Tasmania

## Abstract

Understanding food prices and affordability is crucial for promoting healthy dietary habits and informing policy actions. We assessed changes in the cost and affordability of habitual and recommended healthy diets in Northwest Tasmania from 2021 to 2023. The recommended diet was 16–22% less expensive than the habitual diet during the period. Notably, 60% of the total cost of the habitual diet was spent on discretionary items. The cost of the habitual diet increased by 9% in this period, whereas the cost of the recommended diet increased by only 2%. The habitual diet was unaffordable for households with median gross, minimum wage disposable or welfare-dependent incomes. The recommended diet, however, was affordable for some groups but posed a risk of food stress for those with median gross and minimum wage disposable income and remained unaffordable for those who were welfare dependent. Our findings reveal that adhering to a healthy Australian Dietary Guidelines-recommended diet can be more cost-effective than following a habitual unhealthy diet. However, adopting a healthy diet can be challenging for low-income families. Interventions such as financial support, nutrition education, community gardens and food hubs, as well as price regulation and subsidies for farmers, can help address food insecurity in Northwest Tasmania.

## 1. Introduction

The rapid rise in non-communicable diseases (NCDs), including cardiovascular diseases, cancer, and type 2 diabetes, has severely burdened health systems [1,2,3,4]. Overweight and obesity are well-known risk factors for many NCDs, and obesity is now recognised as a disease [5,6,7,8]. About 2.6 billion adults worldwide are living with overweight or obesity, and projections show that more than 50% of the world’s population will be living with obesity by 2035 [9]. Australia has one of the highest obesity rates in the world, with 67% of adults and 25% of children aged 2–17 years living with overweight or obesity in 2018 [10]. An unhealthy dietary pattern is a major modifiable risk factor for obesity and related NCDs [11,12,13,14]. Australian Dietary Guidelines (ADG) emphasise limiting the intake of food and beverages high in added sugars, saturated fats, sodium and/or alcohol for reduced risk of NCDs [15]. However, dietary choices are influenced by a wide range of factors, including price and affordability [16,17,18]. While the perception that healthy diets cost more than unhealthy diets has long been a barrier to healthy eating, especially among individuals with a lower socio-economic position [19,20,21], recent evidence shows that healthy diets are less expensive than the current habitual unhealthy diet [22]. Despite these findings, less than 4% of Australian adults are reported to follow the healthy ADG-recommended diet [15].

Obtaining robust evidence on food prices and affordability is vital to inform policy action and promote healthy dietary habits. To monitor the cost and affordability of healthy and habitual diets globally, the International Network for Food and Obesity/non-communicable diseases Research, Monitoring and Action Support (INFORMAS) developed a framework, and this approach was adapted in the Australian context to create the Healthy Diets ASAP (Australian Standardised Affordability and Pricing) protocol [23], which has been used to evaluate the cost, cost differential and affordability of habitual (unhealthy) and recommended (healthy, more equitable and sustainable) diets in some Australian regions [24,25]. The recommended diet adheres to the ADGs [23]. In a study conducted in Greater Brisbane, it was found that the price of food and beverages escalated significantly between 2021 and 2022, with the recommended diet becoming 11.5% less affordable for welfare-dependent households in 2022 compared to 2019. The COVID-19-related welfare support of the government provided a brief improvement in affordability in 2020-early 2021 [22].

Food prices in rural and regional areas of Australia have been reported as at least 30% higher than in cities [26]. Northwest (NW) Tasmania is known as the ‘food bowl of the island’ due to its excellent soils and temperate climate, making it a centre of food production [27,28]. However, it also faces significant challenges with the highest prevalence of food insecurity in the state. In addition, Tasmania, including the NW region, has the highest rates of overweight and obesity (70.9%) and associated chronic diseases in the country [29]. Understanding diet pricing outcomes is crucial for informing the development of effective and targeted strategies and policies to improve population dietary habits in NW Tasmania and regional areas worldwide. Therefore, this study aimed to assess changes (2021 versus 2023) in the costs and affordability of recommended healthy and habitual diets in NW Tasmania.

## 2. Materials and Methods

### 2.1. Study Design

Using the Healthy Diets ASAP protocol [23], cross-sectional studies were undertaken in 2021 and 2023 to evaluate the cost, cost differential, and affordability of habitual and recommended (healthy, more equitable and sustainable) diets in NW Tasmania, Australia.

### 2.2. Survey Tools

The Healthy Diets ASAP protocol consists of two diet pricing survey tools: the habitual diet pricing tool and the recommended diet pricing tool. These tools specify the type and quantity of food and beverages per fortnight for a reference household of four (an adult male 31–50 years old, an adult female 31–50 years old, a 14-year-old boy and an 8-year-old girl). The habitual diet was designed based on the most recent data available on the reported nutritional intakes of the Australian population [30], and the recommended diet reflects the ADGs [15]. The recommended diet comprises seven food groups (vegetables and legumes, fruit, wholegrain cereal foods, lean meats and meat alternatives, milk and dairy alternatives, oils, and water), and contains slightly less energy (33,610 kJ/8033 kcal per day for reference household) than the habitual diet (33,869 kJ/8095 kcal). The habitual diet consists of all the items listed in the recommended diet in reduced quantities and additional unhealthy, ‘discretionary’ foods and beverages (including fast foods, alcohol, and artificially sweetened beverages) that are high in saturated fat, added sugar and/or sodium and not necessary for health [15] (Table 1).

### 2.3. Store Locations and Sampling

Data collection for the prices of food and beverages was conducted using a convenience sampling technique. As per the Healthy Diets ASAP protocol, data were collected from two supermarkets (these included one large supermarket such as Coles™ or Woolworths™, and an Independent Grocers Australia-IGA™ outlet), a ‘fast-food’/takeaway outlets, a fish and chips shop, and one alcoholic liquor outlet closest to the geographical centre of each Statistical Area Level 2 (SA2) location in NW Tasmania. SA2 is a spatial unit defined under the Australian Statistical Geography Standard (ASGS) and is small enough to represent a community, allowing data to be analysed at a localised level, leading to more precise and targeted decision making [31]. All the locations selected (Burnie, Circular Head and Devonport) served as SA2 sites and were in quintile 1, i.e., socioeconomically most disadvantaged, based on the Socio-Economic Indexes for Areas (SEIFA) Index of Relative Socio-Economic Disadvantage. Due to the limited number of food outlets in this geographical area, data were also collected from convenience stores and fruit and vegetable shops, which was outside the Healthy Diet ASAP protocol.

### 2.4. Data Collection

Food and beverage prices were collected by trained researchers in accordance with the Healthy Diets ASAP protocol and entered into the Healthy Diets ASAP data collection web portal. Permission to collect data was obtained from the store managers on arrival at each store for data collection. If the specified brand or size was not available, the cheapest brand available in the specified size or the nearest larger size of the specified brand was selected [23]. KA checked the data and discussed any discrepancies with the data collectors the next day. The University of Tasmania Human Research Ethics Committee deemed this project exempt from ethical review based on negligible risk and the collecting of non-identifiable data.

### 2.5. Calculation of Household Income

The Healthy Diets ASAP protocol was used to calculate three types of income. The median gross household income (before taxation) per fortnight in each local government area (LGA) was sourced from the ABS 2021 Census Community Profile [32] and adjusted by the ABS Wage Price Index [33] to calculate the median gross income for 2023. Minimum wage disposable and welfare-dependent incomes for the reference household were calculated based on the assumptions specified in the ASAP protocol, and payment entitlement data were sourced from Services Australia [34].

### 2.6. Data Analysis

The data were cleaned and analysed by MPH and confirmed by KA. According to the Healthy Diets ASAP protocol, any missing price was substituted by the mean price of the same item in other food outlets at the same location and time. The mean prices of other outlets were substituted for the missing items in convenience stores and fruit and vegetable shops. The mean costs of habitual and recommended diets and component food groups were generated from the web-based portal for each SA2 using spreadsheet algorithms in Microsoft Office Excel files. The cost of component food groups was calculated as mean ± standard deviation (SD) and as a proportion of the total cost. Recommended and habitual diet costs were presented as proportions of three different income types to assess affordability. A proportion of ≥30% was considered ‘unaffordable’, and a proportion of 25–30% was considered ‘food stress’ [22]. The results of 2021 were compared to the results of 2023 using paired t-tests. Statistical significance was set at *p* ≤ 0.05. As all the locations selected were in SEIFA quintile 1, the results were reported as for the whole of NW Tasmania.

## 3. Results

### 3.1. Food Outlets Surveyed

In 2021, food prices were collected from food outlets in Burnie, Devonport and Circular Head [35] SA2s (*n* = 17). Data were collected from the same stores in 2023; however, as one food outlet had closed over the two years, the relevant data were collected from another.

### 3.2. The Costs of Recommended and Habitual Diets, and Food Groups in NW Tasmania

In 2021, the mean cost of the recommended diet for the reference household of two adults and two children per fortnight was AUD 723.66 (Table 2). The cost of the habitual diet per fortnight (AUD 865.75) was AUD 142.09 more than the recommended diet (*p* = 0.016). In 2023, the costs of recommended and habitual diets were AUD 738.42 and AUD 945.99, respectively, further increasing the cost differential (AUD 207.58, *p* < 0.001). In both years, nearly 60% (AUD 513.17 in 2021 and AUD 572.75 in 2023) of the total cost of the habitual diet was spent on discretionary items (approximately 20% on takeaway foods, 13% on alcohol and 27% on other discretionary items). In fact, the habitual diet was still more expensive than the recommended diet, even without the cost of alcohol (AUD 748.69 in 2021 and AUD 823.14 in 2023).

Between 2021 and 2023, the cost of the recommended diet increased by only 2.04%, from AUD 723.66 to AUD 738.42 (*p* = 0.205). The cost of the habitual diet increased significantly (by 9.27%) over the same period, from AUD 865.75 to AUD 945.99 (*p* = 0.032). In the recommended diet, the food groups that showed significant cost increases were vegetables and legumes (12.17% increase, *p* = 0.016) and lean meat, poultry, fish, eggs, and alternatives (7.61% increase, *p* = 0.013). While the cost of water (3.13% to 2.47%), fruit (14.06% to 13.10%) and grain foods (17.87% to 16.15%) in the recommended diet reduced from 2021 to 2023, the reductions were not significant. In the habitual diet, three healthy food groups showed significant price increases: vegetables and legumes (12.79% increase, *p* = 0.016); lean meat, poultry, fish, eggs and alternatives (8.40% increase, *p* = 0.014); and milk, yoghurt, cheese and alternatives (7.67% increase, *p* = 0.013). In the habitual diet, a significant price increase was observed in artificially sweetened soft drinks (14.24% increase, *p* = 0.023). The prices of take-away foods and alcoholic drinks were also substantially increased; however, significance tests could not be performed as the prices were based on data collected from a single shop each year. The costs of water and grain food components of the habitual diet also decreased from 2021 to 2023, although the changes were not significant.

### 3.3. Affordability of the Recommended and Habitual Diets in Northwest Tasmania

In 2021 and 2023, the median gross income per fortnight (AUD 2500.00 and AUD 2625.00, respectively) was about 6% less than the minimum wage income (AUD 2679.10 and AUD 2806.31, respectively) and about 32–33% higher than the welfare income (AUD 1873.27 and AUD 1986.19, respectively; Table 3).

A reference household on a median gross income would have experienced food stress when purchasing the recommended diet, which cost about 28.95% of their income in 2021 and 28.13% in 2023. However, the habitual diet was unaffordable for them, and the proportion of income they would have to spend on purchasing the habitual diet increased from 34.63% to 36.04%. A reference household on the minimum wage would have experienced a similar situation, with following a recommended diet causing food stress, while the habitual diet was unaffordable at the 30% affordability threshold. Both the recommended and habitual diets were unaffordable for those who were welfare-dependent in 2021 and 2023, but the proportion of income they would have had to spend on the habitual diet (46.22% and 47.63%, respectively) was much higher than that for the recommended diet (38.63% and 37.18%, respectively).

## 4. Discussion

This study employed the Healthy Diets ASAP protocol [23] to evaluate the cost differential and affordability of recommended and habitual diets in NW Tasmania from 2021 to 2023. The recommended diet was 16–22% less expensive than the habitual diet during this period. Approximately 60% of the total cost of the habitual diet was spent on discretionary items. From 2021 to 2023, the cost of the recommended healthy diet increased by 2.04%, while the cost of the habitual diet increased by 9.27%—nearly five times that of the former. When using an arbitrary benchmark of 30% as diet affordability, the habitual diet was unaffordable for households with median gross, minimum wage disposable or welfare-dependent incomes, indicating a high likelihood of food insecurity in the area during the period. In contrast, the recommended diet was affordable for those receiving a median gross income or a minimum wage disposable income, but these households were at risk of food stress. However, the recommended diet was not affordable for those households who were welfare dependent.

Our findings are in line with the results of other Healthy Diets ASAP studies conducted during 2017–2021, which demonstrated that the habitual unhealthy diet was 14–23% more expensive than the ADG-recommended healthy diet in various regions across Australia, including Queensland [25], Brisbane [22], Sydney and Canberra [24]. The cost advantage of the recommended diet compared to the habitual diet can be attributed, in part, to Australia’s differential taxation to promote healthy diets, as basic healthy foods are exempted from the 10% Goods and Services Tax (GST). The exclusion of alcohol from the recommended diet also contributes to its relative affordability. However, even without the cost of alcohol factored in, our data revealed that the habitual diet remained less affordable in NW Tasmania (AUD 748.69 in 2021 and AUD 823.14 in 2023) compared to the recommended diet (AUD 723.66 in 2021 and AUD 738.42 in 2023).

In a previous survey in March-April 2014, the cost and affordability of healthy food items were assessed in Tasmania using the Healthy Food Access Basket (HFAB) tool [36] based on the Victorian Health Food Basket protocol [37]. HFAB included 44 foods which were consistent with the Australian Guide to Healthy Eating (AGHE) [38], and it has been widely used to measure healthy food prices across Australia. The mean cost of the HFAB for two adults and two children for two weeks was AUD 435.50 in NW Tasmania in 2014 [36]. Compared to the cost of the recommended diet in 2023 (AUD 738.42), there has been approximately a 70% increase in the cost of a healthy diet over the 9-year period from 2014 to 2023. A reference family of two adults and two children in 2014 with a fortnightly government assistance income (AUD 1353.42) would have spent approximately 32.18% of their income to purchase HFAB [36].

Zorbas et al. [39] conducted a cross-sectional study to collect publicly available food and beverage price data in Australia using the Healthy Diets ASAP protocol. In 2019, out of all states and territories, the habitual diet was cheapest in Tasmania (AUD 732.85), compared to Northern Territory (AUD 764.68), where it was most expensive. The recommended healthy diet was cheaper than the habitual diet in all states and territories, including Tasmania (AUD 590.75). For a reference family of four with a median gross household income in Tasmania in 2019, the habitual diet costing 31.51% of the income was not affordable, and the recommended diet was at the food stress threshold (25.09%), whereas both diets were affordable and required spending a lower proportion of income in other states [39]. Our results for NW Tasmania are consistent with the results of Zorbas et al. [39] and indicate that the proportion of median gross income a reference family needed to spend on the habitual diet further increased from 2021 to 2023 (34.63% to 36.04%), while the proportion needed for the recommended diet slightly decreased (28.95% to 28.13%). However, the median gross income represents income before tax, which means that the actual income is lower, and hence the diets may be even more unaffordable than noted here.

More recently, the Tasmania Project Cost of Living Survey reported that one in two (51%) Tasmanian households experienced food insecurity in September–October 2022, nearly double the rate reported in May 2021 (27%). In addition, we have generally observed several closures of food outlets in NW Tasmania from 2021 to 2023, probably due to economic effects stemming from the pandemic, including reduced tourism and high labour costs driven by inflation. This may add to the burden of food costs, as there can be additional expenses associated with travelling long distances to other food outlets.

A Healthy Diets ASAP study [22] reported that diet prices increased in Brisbane from 2019 to 2021 due to continuing effects of COVID-19, such as reduced workforce and supply chain disruptions and the wild bushfires of 2019–2020. From 2019 to 2022 in Brisbane, the habitual diet cost increased by 19.5%, whereas the recommended diet cost increased by 17.7%. However, from 2021 to 2022, the relative cost increase of the habitual diet was lower than that of the recommended diet (7.7% vs. 12.6%, respectively), which is quite the opposite of our findings. From 2021 to 2023 in NW Tasmania, the relative cost increase of the habitual diet was greater than the recommended diet (9% vs. 2%). This could be attributed to the rise in packaging and processing costs in Australia during the first quarter of 2023 [33]. Additionally, the freight transportation costs to Tasmania are higher, as there is no option of transporting goods by rail or road from other states. The diet costs in Greater Brisbane in 2021 (recommended: AUD 647.18; habitual: AUD 801.13) were found to be lower than in NW Tasmania (recommended: AUD 723.66; habitual: AUD 865.75). The higher prices observed in NW Tasmania in 2021 could be a result of food supply chain disruptions due to strong border restrictions between May 2020 and November 2021. Moreover, high fuel and fertiliser costs due to the Russian invasion of Ukraine [40] and elevated global and national inflation [41] may have further contributed to the price increase from 2021 to 2023 in Tasmania.

Consistent with other Healthy Diets ASAP findings [24], a reference family of four would spend around 60% of their food budget on unhealthy discretionary items, with a large proportion expended on takeaways (approximately 20%). While the increasing density of food outlets, busy lifestyles, advertising, and marketing have a role in the increasing popularity of out-of-home foods, food choice can result from poor food literacy or low cooking skills [42,43,44]. With regular consumption of takeaways being identified as a critical driver of the obesity epidemic [45,46,47], further investigations are needed to understand the reasons for regular out-of-home food consumption and programs to strengthen the knowledge of its unfavourable nutritional content.

Addressing the issue of making food more affordable can be tackled through a socio-ecological model [48] which operates at different levels to address the problem comprehensively. Firstly, at the micro level (individual and household), it is important to acknowledge that welfare payments often do not provide sufficient support for vulnerable individuals and families to meet their basic needs. Rising living costs, particularly for essential goods like healthy food, pose significant financial challenges for welfare recipients seeking nutritious meals and overall well-being. Solutions include offering financial support, food subsidies, and providing food and nutrition education to individuals and households, for example, through schools and council-based community programs. Maintaining the GST exemption for basic healthy food items can relieve some financial burdens on low-income households, ensuring that essential nutritious foods remain affordable and accessible to those in need. Addressing the inadequacy of welfare payments and safeguarding the GST exemption for basic healthy food goes beyond economic policy; it is a moral obligation to prioritise the health and well-being of all citizens and strive for a more inclusive and equitable society. As housing and utility costs increase, more of the household budget is allocated to cover these expenses, leaving less room for discretionary spending on food. This can lead to trade-offs between essential expenses and accessing nutritious meals. Secondly, at the meso level (community and local), community-led initiatives can play a vital role, such as establishing community gardens, school gardens, farmers’ markets, coops, food hubs, and social enterprises. Thirdly at the macro level (national and global), comprehensive policy solutions that consider the interconnectedness of housing, utilities, fuel, and food affordability are crucial. Such initiatives can ensure that everyone has access to affordable, nutritious food and can maintain a decent standard of living [49,50].

Understanding people’s attitudes about making dietary changes for better health is crucial for public health initiatives. An individual’s attitude toward changing dietary habits can be influenced by a combination of factors [51]. One critical determinant is awareness and knowledge about the health consequences associated with dietary choices; individuals with a greater understanding of the nutritional value of foods tend to express positive attitudes toward adopting recommended diet plans [52]. Furthermore, perceptions and emotional responses influenced by personal beliefs and life experiences can influence dietary choices. Those who perceive healthier options to be flavourful and enjoyable are more inclined to include them in their eating patterns [53]. Additionally, social norms, prevailing cultural practices, traditions, and familial influences can also exert a substantial impact on dietary choices. When the prevailing attitude within their social and cultural contexts supports healthy eating, individuals are more likely to adopt healthy dietary practices [54,55]. Personalised approaches and tailored interventions that consider an interplay of these factors are essential for shifting attitudes toward healthy eating habits. However, access to a food environment that provides an affordable healthy diet can assist a household in implementing such attitudes into practice [56]. 

A strength of our study is using a standardised method to evaluate diet prices and affordability, which helps compare results from other locations and over time. The in-built limitations of the Healthy Diets ASAP methodology, including the notion that there is no home food production and the assumptions used for calculating household incomes, have been reported elsewhere [23]. The data collection locations were selected using a convenience sampling technique and covered only three SA2s in NW Tasmania (Burnie, Circular Head and Devonport) which did not include other remote locations in the area. Further research is needed to understand the diet costs and affordability in more remote communities.

In conclusion, a healthy ADG-recommended diet can be more affordable than the habitual diet. Nevertheless, adhering to a healthy diet poses significant challenges for families with a limited household income. The necessity to allocate substantial proportions of income (>30%) to food expenses can lead to household food insecurity, underscoring the urgency to tackle this problem at multiple levels. The study highlights the importance of effective strategies, such as policy development, nutrition education campaigns and tailoring interventions to individuals’ needs, for promoting healthy dietary habits in NW Tasmania and similar settings.

## Figures and Tables

**Table 1:** Food and drinks included in the data collection.

Recommended Diet *	Habitual Diet *
Water (bottled) Fruit: apples, bananas, oranges Vegetables: potatoes, broccoli, white cabbage, iceberg lettuce, onions, carrots, pumpkins, tomatoes, sweetcorn (canned), four-bean mix (canned), diced tomatoes (canned), baked beans (canned), frozen mixed vegetables, frozen peas, salad vegetables in sandwich Grain (cereals): wholegrain cereal biscuits (Weet-bix™), rolled oats, cornflakes, wholemeal bread, white bread, white rice, white pasta, dry water crackers, bread in sandwich Lean meats and alternatives: beef mince and steak, lamb chops, cooked chicken, tuna (canned), eggs, peanuts (unsalted), meat in sandwich Milk, yoghurt, and cheese: cheddar cheese (full-fat, reduced-fat), milk (full-fat, reduced-fat), yoghurt (full-fat plain, reduced-fat flavoured) Unsaturated oils and spreads: olive oil, sunflower oil, canola (margarine)	Healthy foods and drinks as per the seven food groups in the ‘Recommended diet’ column in reduced amounts, reflecting reported intakes Artificially sweetened beverages Discretionary (unhealthy) foods and drinks: ○ Drinks: sugar-sweetened beverages ○ Cereals, snacks, and desserts: muffins, sweet biscuits, savoury crackers, confectionery, chocolate, potato crisps, muesli bars, mixed nuts (salted), ice cream, fruit salad (canned in juice) ○ Processed meats: beef sausages, ham ○ Spreads, sauces, condiments, and ingredients: butter, tomato sauce, salad dressing, white sugar ○ Convenience meals: frozen lasagne, chicken soup (canned), frozen fish fillet (crumbed), instant noodles, meat, and vegetable stew (canned) ○ Fast food: pizza, meat pie, hamburger, potato chips/fries ○ Alcohol: beer (full strength), white wine (sparkling), red wine, whisky

* The recommended and habitual diets were defined as in the Healthy Diets ASAP (Australian Standardised Affordability and Pricing) protocol. The recommended diet has been developed to comply with National Health and Medical Research Council (2013) Australian Dietary Guidelines. Canberra: National Health and Medical Research Council.

**Table 2:** Costs of recommended and habitual diets and component food groups for the reference household per fortnight in Northwest Tasmania in 2021 and 2023.

Food Groups	2021						2023					
	Recommended			Habitual			Recommended			Habitual		
	Mean	(SD)	Proportion of Total Cost (%)	Mean	(SD)	Proportion of Total Cost (%)	Mean	(SD)	Proportion of Total Cost (%)	Mean	(SD)	Proportion of Total Cost (%)
**Water**	22.66	(3.31)	3.13%	22.66	(3.31)	2.62%	18.24	(1.21)	2.47%	18.24	(1.21)	1.93%
**Fruit**	101.78	(5.57)	14.06%	57.57	(5.04)	6.65%	96.77	(3.38)	13.10%	64.46	(3.13)	6.81%
**Vegetables and legumes**	116.99	(7.89)	16.17%	46.58	(2.64)	5.38%	**131.22**	(7.07)	17.77%	**52.54**	(2.3)	5.55%
**Grain foods (cereals)**	129.28	(2.47)	17.87%	52.45	(1.4)	6.06%	119.25	(4.76)	16.15%	50.31	(4.91)	5.32%
**Lean meat, poultry, fish, eggs, and alternatives**	223.94	(7.01)	0.31%	116.26	(3.78)	13.43%	**240.99**	(3.77)	32.64%	**126.03**	(3.46)	13.32%
**Milk, yoghurt, cheese, and alternatives**	119.05	(4.22)	16.45%	55.56	(0.99)	6.42%	120.86	(5.78)	16.37%	**59.81**	(2.3)	6.32%
**Unsaturated oils and spreads**	9.96	(0.26)	1.38%	1.49	(0.2)	0.17%	11.09	(1.01)	1.50%	1.85	(0.11)	0.20%
**Sugar-sweetened soft drinks**				29.98	(5.94)	3.46%				33.13	(3.44)	3.50%
**Artificially sweetened soft drinks**				5.77	(0.86)	0.67%				**6.59**	(0.69)	0.70%
**Discretionary choices—other**				194.35	(26.92)	22.45%				215.49	(2.04)	22.78%
Take-away foods *				166.01		19.18%				194.68		20.58%
Alcoholic drinks *				117.06		13.52%				122.86		12.99%
**Total**	723.66	(12.26)		865.75	(39.26)		738.42	(16.95)		**945.99**	(14.67)	

Means (SDs) of the recommended and habitual diet costs are given in AUD. Bold font denotes statistical significance at *p* < 0.05 in the change of cost from 2021 to 2023; SD: standard deviation; reference household: two parents with two children (adult male (between 31 and 50 years), adult female (between 31 and 50 years), 14-year-old boy and 8-year-old girl). * Costs of take-away foods and alcoholic drinks were calculated with data from a single shop for each year; hence, SD calculations or paired t-tests could not be performed.

**Table 3:** Household incomes of the reference household per fortnight and affordability of the recommended and habitual diets.

Income Type	2021			2023		
	Income	Proportion for Recommended Diet	Proportion for Habitual Diet	Income	Proportion for Recommended Diet	Proportion for Habitual Diet
Median gross	2500.00	28.95%	34.63%	2625.00	28.13%	36.04%
Minimum wage disposable	2679.10	27.01%	32.31%	2806.31	26.31%	33.71%
Welfare dependent	1873.27	38.63%	46.22%	1986.19	37.18%	47.63%

Income is given in AUD. Affordability is calculated as the cost of the diet divided by household income; a proportion ≥30% is considered ‘unaffordable’, and a proportion 25–30% is considered ‘food stress’; reference household: two parents with two children (adult male (between 31 and 50 years), adult female (between 31 and 50 years), 14-year-old boy and 8-year-old girl).

## Data Availability

Data are available upon request from the authors.
